# Ferro- and Antiferromagnetic Interactions in Oxalato-Centered Inverse Hexanuclear and Chain Copper(II) Complexes with Pyrazole Derivatives

**DOI:** 10.3390/molecules26092792

**Published:** 2021-05-10

**Authors:** Isabel Castro, M. Luisa Calatayud, Marta Orts-Arroyo, Nicolás Moliner, Nadia Marino, Francesc Lloret, Rafael Ruiz-García, Giovanni De Munno, Miguel Julve

**Affiliations:** 1 Instituto de Ciencia Molecular (ICMol), Universitat de València, 46980 Paterna, Spain; marialuisacalatayud@gmail.com (M.L.C.); martaorts.a@gmail.com (M.O.-A.); nmoliner@gmail.com (N.M.); francisco.lloret@uv.es (F.L.); rafael.ruiz@uv.es (R.R.-G.); 2 Dipartimento di Chimica e Tecnologie Chimiche, Università della Calabria, 87036 Rende, Italy

**Keywords:** copper, oxalato, pyrazole, crystal structure, inverse coordination chemistry, polynuclear complexes, coordination polymers, magnetic properties

## Abstract

Two novel copper(II) complexes of formulas {[Cu(4-Hmpz)_4_][Cu(4-Hmpz)_2_(µ_3_-ox-*κ*^2^*O*^1^,*O*^2^:*κO*^2′^:*κO*^1′^)(ClO_4_)_2_]}*_n_* (**1**) and {[Cu(3,4,5-Htmpz)_4_]_2_[Cu(3,4,5-Htmpz)_2_(µ_3_-ox-*κ*^2^*O*^1^,*O*^2^:*κO*^2′^:*κO*^1′^)(H_2_O)(ClO_4_)]_2_[Cu_2_(3,4,5-Htmpz)_4_(µ-ox-*κ*^2^*O*^1^,*O*^2^:*κ*^2^*O*^2′^,*O*^1′^)]}(ClO_4_)_4_·6H_2_O (**2**) have been obtained by using 4-methyl-1*H*-pyrazole (4-Hmpz) and 3,4,5-trimethyl-1*H*-pyrazole (3,4,5-Htmpz) as terminal ligands and oxalate (ox) as the polyatomic inverse coordination center. The crystal structure of **1** consists of perchlorate counteranions and cationic copper(II) chains with alternating bis(pyrazole)(µ_3_-*κ*^2^*O*^1^,*O*^2^:*κO*^2′^:*κO*^1′^-oxalato)copper(II) and tetrakis(pyrazole)copper(II) fragments. The crystal structure of **2** is made up of perchlorate counteranions and cationic centrosymmetric hexanuclear complexes where an inner tetrakis(pyrazole)(µ-*κ*^2^*O*^1^,*O*^2^:*κ*^2^*O*^2′^,*O*^1′^-oxalato)dicopper(II) entity and two outer mononuclear tetrakis(pyrazole)copper(II) units are linked through two mononuclear aquabis(pyrazole)(µ_3_-*κ*^2^*O*^1^,*O*^2^:*κO*^2′^:*κO*^1′^-oxalato)copper(II) units. The magnetic properties of **1** and **2** were investigated in the temperature range 2.0–300 K. Very weak intrachain antiferromagnetic interactions between the copper(II) ions through the µ_3_-ox-*κ*^2^*O*^1^,*O*^2^:*κO*^2′^:*κO*^1′^ center occur in **1** [*J* = −0.42(1) cm^−1^, the spin Hamiltonian being defined as ***H*** = −*J∑**S***_1,i_ · ***S***_2,i**+1**_], whereas very weak intramolecular ferromagnetic [*J* = +0.28(2) cm^−1^] and strong antiferromagnetic [*J’* = −348(2) cm^−1^] couplings coexist in **2** which are mediated by the µ_3_-ox-*κ*^2^*O*^1^,*O*^2^:*κO*^2′^:*κO*^1′^ and µ-ox-*κ*^2^*O*^1^,*O*^2^:*κ*^2^*O*^2′^,*O*^1′^ centers, respectively. The variation in the nature and magnitude of the magnetic coupling for this pair of oxalato-centered inverse copper(II) complexes is discussed in the light of their different structural features, and a comparison with related oxalato-centered inverse copper(II)-pyrazole systems from the literature is carried out.

## 1. Introduction

The term inverse coordination chemistry (ICC) was coined by Haiduc to describe an emerging research area in the field of coordination chemistry, where the bridging ligand plays the role of coordination center surrounded by transition metal ions which can be further coordinated to other co-ligands acting as additional bridging or terminal donors [1,2,3,4,5,6,7,8]. ICC provides new concepts for the structural description and future perspectives in the design and synthesis of inverse polynuclear complexes (IPCs) and inverse coordination polymers (ICPs) with tailor-made nuclearity and dimensionality (*n*D, *n* = 1−3) or topology [1]. The structural directing role of the bridging ligand as a non-metallic inverse coordination center (centroligand) is the cornerstone of the ICC viewpoint. Moreover, it is ultimately responsible for the magnetic properties of a unique class of IPCs and ICPs with paramagnetic 3d–5d metal ions that behave as molecular nanomagnets and molecule-based magnets [9,10,11,12,13,14,15,16,17,18,19,20,21,22,23,24]. They include discrete metal-fluoride rings [25,26,27,28], metal-oxo clusters [29,30,31], and metal-cyanide polyhedra [32], as well as a variety of chains, layers, and different networks with cyanide [33,34] and polycyanocarbon anion radicals [35,36].

**Scheme 1:**
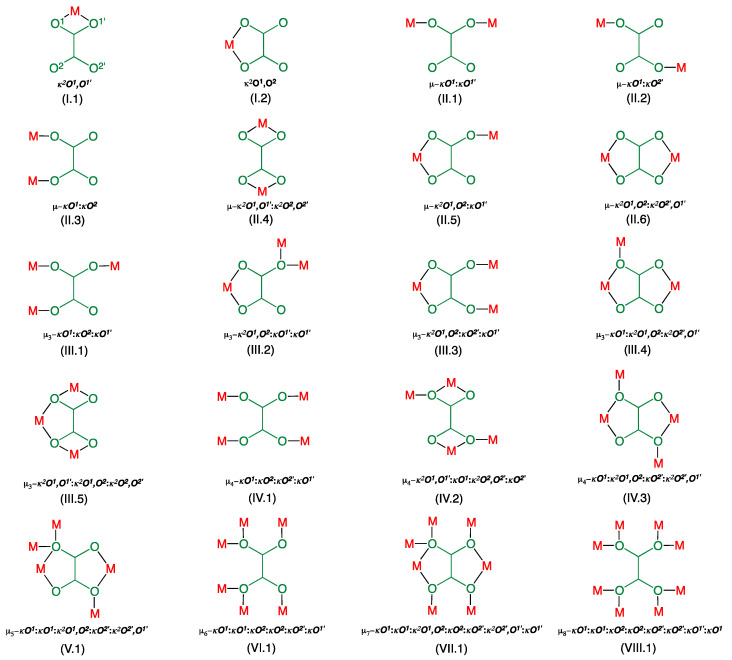
Coordination modes of the oxalate dianion in its metal complexes.

Oxalate (C_2_O_4_^2−^, ox) is the simplest dicarboxylate dianion, and it constitutes a very versatile polyatomic “maverick” ligand because of the great number of coordination modes that it can adopt in its complexes (Scheme 1) [8]. For instance, it can exhibit two chelating bidentate coordination modes as a terminal ligand to render traditional (Werner-type) mononuclear metal complexes with head-on or side-on configuration (**I.1** and **I.2** in Scheme 1). Oxalate is also able to function as a centroligand toward several metal ions, from two up to eight (**II.1-6**, **III.1-5**, **IV.1-3**, **V.1**, **VI.1**, **VII.1**, and **VIII.1** in Scheme 1), giving rise to diverse IPCs and ICPs as unique examples of molecular nanomagnets and molecule-based magnets, respectively [37,38,39]. In fact, oxalate is a well-known good mediator of magnetic interactions between two copper(II) ions separated by relatively long intermetallic distances [40]. The strength and nature of the magnetic coupling, either ferro- or antiferromagnetic, eventually depend on the coordination mode of the oxalate center and on the geometry at the involved copper(II) ions [41,42,43,44,45,46,47,48,49,50].

Herein, we focus on the synthesis and magneto-structural characterization of two novel oxalato-centered inverse copper(II) complexes of formulas {[Cu(4-Hmpz)_4_][Cu(4-Hmpz)_2_(µ_3_-ox-*κ*^2^*O*^1^,*O*^2^:*κO*^2′^:*κO*^1′^)(ClO_4_)_2_]}*_n_* (**1**) and {[Cu(3,4,5-Htmpz)_4_]_2_[Cu(3,4,5-Htmpz)_2_(µ_3_-ox-*κ*^2^*O*^1^,*O*^2^:*κO*^2′^:*κO*^1′^)(H_2_O)(ClO_4_)]_2_[Cu_2_(3,4,5-Htmpz)_4_(µ-ox-*κ*^2^*O*^1^,*O*^2^:*κ*^2^*O*^2′^,*O*^1′^)]}(ClO_4_)_4_·6H_2_O (**2**) (4-Hmpz = 4-methyl-1*H*-pyrazole and 3,4,5-Htmpz = 3,4,5-trimethyl-1*H*-pyrazole). Our goal is to investigate the influence of the steric and/or electronic ligand effects on the structure and magnetic properties of these oxalato-centered ICP and IPC with polymethyl-substituted pyrazole derivatives as terminal ligands.

## 2. Experimental

### 2.1. Materials

Oxalic acid, 4-Hmpz, 3,4,5-Htmpz, copper(II) perchlorate hexahydrate, and triethylamine were purchased from commercial sources and they were used as received without any further purification.

### 2.2. Preparations of the Complexes

#### 2.2.1. {[Cu(4-Hmpz)_4_][Cu(4-Hmpz)_2_(µ-ox-*κ*^2^*O*^1^,*O*^2^:*κO*^2′^:*κO*^1′^)(ClO_4_)_2_]}_n_ (**1**)

A water/methanol (1:1 *v*/*v*) solution (30 mL) of 4-Hmpz (0.245 g, 3.0 mmol) was poured into an aqueous solution (20 mL) of copper(II) perchlorate hexahydrate (0.370 g, 1.0 mmol). Then, an aqueous solution (10 mL) containing oxalic acid (0.045 g, 0.5 mmol) and triethylamine (0.2 mL, 1.0 mmol) was then added to the previous mixture and the whole was kept under continuous stirring for 20 min at room temperature and filtered to remove any remaining small particle. Single crystals of **1** as deep blue prisms were grown from the resulting blue solution after some days by slow evaporation at room temperature. They were collected and dried on filter paper. Yield: 0.28 g, 55%. Anal. Calcd. for C_26_H_36_Cl_2_Cu_2_N_12_O_12_ (MW = 906.6 g mol^−1^): C, 34.4; H, 4.0; N, 18.5%. Found: C, 34.5; H, 3.6; N, 17.4%. IR (KBr, cm^−1^): 3357 m, 3279 w [υ(N–H) from 4-Hmpz], 3125 m, 2987 m, 2934 w, 2876 w [υ(C–H) from 4-Hmpz], 1675 s, 1652(sh) [υ_as_(C=O) from ox], 793 m [υ(O–C–O], 1438 m [υ(C=N) from 4-Hmpz], 1064 s, 965 w, and 622 m [υ(Cl–O) from perchlorate].

#### 2.2.2. {[Cu(3,4,5-Htmpz)_4_]_2_[Cu(3,4,5-Htmpz)_2_(µ_3_-ox-*κ*^2^*O*^1^,*O*^2^:*κO*^2′^:*κO*^1′^)(H_2_O)(ClO_4_)]_2_ [Cu_2_(3,4,5-Htmpz)_4_(µ-ox-*κO*^1^,*O*^2^:*κ*^2^*O*^2′^,*O*^1′^)]}(ClO_4_)_4_·6H_2_O (**2**)

3,4,5-Htmpz (0.275 g, 2.5 mmol) dissolved in a water/methanol (1:1 *v*/*v*) solvent mixture (30 mL) was added to an aqueous solution (20 mL) of copper(II) perchlorate hexahydrate (0.370 g, 1.0 mmol) under continuous stirring. An aqueous solution (10 mL) of oxalic acid (0.045 g, 0.5 mmol) and triethylamine (0.2 mL, 1.0 mmol) was then poured into the above solution and the whole was stirred for 20 min at room temperature. The resulting blue solution was filtered to remove any remaining small particle and allowed to evaporate in a hood under ambient conditions. X-ray suitable deep blue-greenish prisms of **2** were formed after a few days. They were collected by filtration and dried on filter paper. Yield: 0.29 g, 65%. Anal. Calcd. for C_102_H_176_Cl_6_Cu_6_N_32_O_44_ (MW = 3148.68 g mol^−1^): C, 38.9; H, 5.6; N, 14.2%. Found: C, 38.1; H, 5.7; N, 14.4%. IR (KBr, cm^−1^): 3505 m [υ(O–H from water], 3355 m, 3221 w [υ(N–H from 3,4,5-Htmpz], 2926 w, 2867 w [υ(C–H) from 3,4,5-Htmpz], 1673(sh), 1662 s, 1645(sh) [υ_as_(C=O) from ox], 796 m [υ(O–C–O], 1447 m [υ(C=N) from 3,4,5-Htmpz], 1091 s, 953 w, and 633 m [υ(Cl–O, from perchlorate].

### 2.3. Physical Techniques

Elemental analyses (C, H, N) were performed by the Servei Central de Suport a la Investigació Experimental de la Universitat de València. FT-IR spectra were recorded on a Nicolet-5700 spectrophotometer as KBr pellets. Powder X-ray diffraction (XPRD) patterns of powdered crystalline samples were collected at room temperature on a D8 Avance A25 Bruker diffractometer by using graphite-monochromated Cu-Kα radiation (*λ* = 1.54056 Å). Variable-temperature (2.0–300 K) direct current (dc) magnetic susceptibility measurements were carried out with a SQUID magnetometer under applied fields of 5.0 kOe (*T* > 20 K) and 250 Oe (*T* < 20 K) to prevent for saturation effects at low temperature. The experimental magnetic susceptibility data were corrected for the diamagnetic contributions of the constituent atoms and the sample holder (a plastic bag), as well as for the temperature-independent paramagnetism (tip) of the copper(II) ion (60 × 10^−6^ cm^3^·mol^−1^).

### 2.4. Crystallographic Data Collection and Refinement

X-ray diffraction data on single crystals of **1** and **2** were collected at room temperature with Bruker D8 Venture with PHOTON II detector (**1**) and Bruker-Nonius X8-APEXII CCD area detector (**2**) diffractometers by using graphite-monochromated Mo-Kα radiation (λ = 0.71073 Å). The data were processed through the SAINT [51] reduction and SADABS [52] absorption software. The structures were solved using direct methods and subsequently completed by Fourier recycling using the SHELX-2018 software package [53,54], then refined by the full-matrix least-squares refinements based on *F*^2^ with all observed reflections. All non-hydrogen atoms of **1** and **2** were refined anisotropically. The hydrogen atoms of the polymethyl-substituted pyrazole ligands in **1** and **2** were placed on calculated positions and refined using a riding model. The hydrogen atoms of the water molecules in **2** were initially located from the Fourier difference map and a few suitable hydrogen bonds were found; eventually, they were excluded from the refinement as their location was losing reliability after some refinement cycles, coming too close to other atoms in the structures, probably due to the confined space occupied by the water molecules in the structure. The perchlorate counterions in **2** were found to be involved in somewhat standard disorders, and almost all of the oxygen atoms were modeled over two sites. These disorders were refined freely within SHELXL, using similarity restraints on 1,2- and 1,3-distances as well as rigid-bond restraints, while constraining the sum of the occupancies to unit [54]. The final geometrical calculations and graphical manipulations for **1** and **2** were performed using the XP utility within SHELX [53] and the CrystalMaker program [55]. Crystallographic data have been deposited at the Cambridge Crystallographic Data Centre with CCDC reference numbers 2076622 (**1**) and 2076621 (**2**). A summary of the crystallographic data and structure refinement for **1** and **2** is given in Table 1. Selected bond distances and angles and hydrogen bonds for **1** and **2** are listed in Tables S1–S4 (see Supplementary Materials).

## 3. Results and Discussion

### 3.1. Synthesis and General Characterization

**1** and **2** were prepared by the reaction of the in situ generated oxalato dianion (mixture of H_2_ox and triethylamine in a 1:2 molar ratio) with copper(II) perchlorate and the corresponding pyrazole derivative [3,4,5-Htmpz (**1**) and 4-Hmpz (**2**)] under nominal 1:3 (**1**) and 1:2.5 (**2**) copper(II) to pyrazole molar ratios in water/methanol solvent mixtures at room temperature (see the Experimental Section). The chemical identity of **1** and **2** was confirmed by elemental analyses (C, H, N) and FT-IR spectroscopy (Figure S1 in the Supplementary Material), and it was further supported by powder X-ray diffraction (PXRD). Indeed, the PXRD patterns of **1** and **2** coincide with the calculated ones from the single-crystal X-ray analyses (Figure S2 in the Supplementary Material) confirming the purity of the bulk material.

The occurrence of the N–H stretching vibration in the wavenumber range 3200–3360 cm^−1^ [υ(N–H)], together with several weak absorptions between 3125 and 2860 cm^−1^ [υ(C–H)] in the infrared spectra of **1** and **2** support the presence of the methyl-substituted pyrazole derivatives in them. Strong and broad absorption peaks at 3550–3400 cm^−1^ [υ(O–H)] for **2** are indicative of the occurrence of water molecules involved in hydrogen bonding [56]. The strong absorptions at 1660–1680 cm^−1^ [υ_as_(C=O)] and medium intensity peaks at 790–800 cm^−1^ [υ(O–C–O] for **1** and **2** are a diagnostic of the presence of bridging oxalate [56]. Finally, the set of strong (υ_3_), weak (υ_1_), and medium (υ_4_) intensity peaks at 1064, 965, and 622 cm^−1^ (**1**)/1091, 953, and 633 (**2**) [υ(Cl–O)] suggest the occurrence of weakly coordinated anionic perchlorate groups [57]. All of these spectroscopic features were confirmed by the single-crystal X-ray analysis.

In the following, a comprehensive description of the crystal structures of **1** and **2** is given in the framework of the ICC approach, a particular attention being paid to the elucidation of the possible magneto-structural correlations along this oxalate-centered copper(II)-pyrazole family.

### 3.2. Description of the Structures

#### 3.2.1. {[Cu(4-Hmpz)_4_][Cu(4-Hmpz)_2_(µ_3_-ox-*κ*^2^*O*^1^,*O*^2^:*κO*^2′^:*κO*^1′^)(ClO_4_)_2_]}_n_ (**1**)

**Figure 1:**
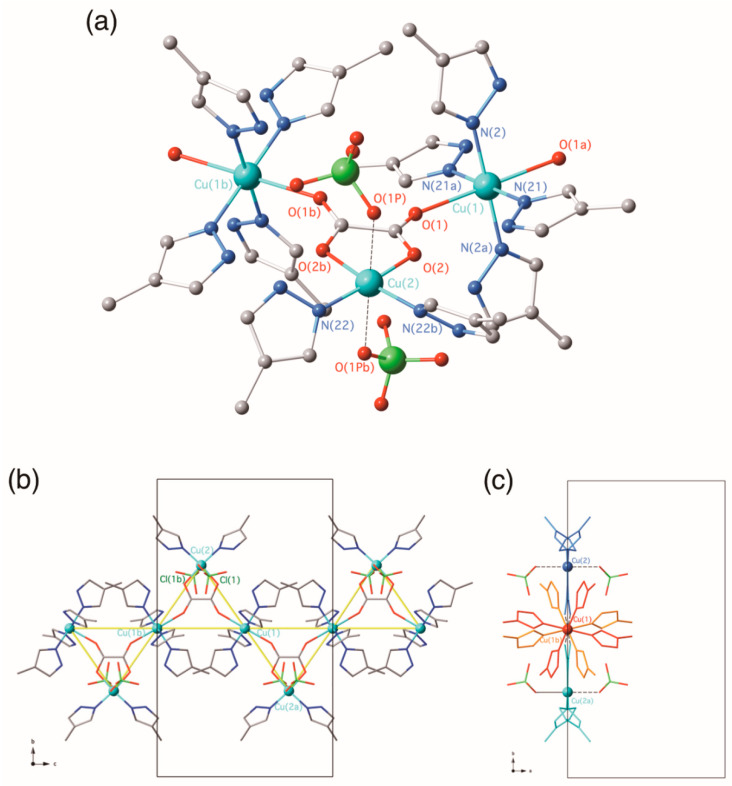
(**a**) Perspective view of a fragment of the cationic copper(II) chain of **1** with the atom-numbering scheme of the donor set at each copper site [symmetry code: (a) 1 − *x*, − *y*, − *z*; (b) 1 − *x*, *y*, 1/2 − *z*]. Hydrogen atoms are omitted for clarity. Selected bond lengths (Å): Cu(1)–N(2) = 2.011(2), Cu(1)–N(21) = 2.045(2), Cu(1)–O(1) = 2.3766(14), Cu(2)–N(22) = 1.964(2), Cu(2)–O(2) = 1.9585(14), and Cu(2)–O(1P) = 2.496(4) (data from Table S1); (**b**,**c**) projection views of the inverse oxalato-centered copper(II) chain of branch-type topology along the crystallographic *a* and *c* axes, respectively. The weak axial coordinative bonds with the perchlorate anions are depicted by black dotted lines. The intramolecular Cu···Cu separations through the µ_3_-ox-*κ*^2^*O*^1^,*O*^2^:*κO*^2′^:*κO*^1′^ centroligands within the vertex-shared triangular copper(II) entities are evidenced by thick yellow solid lines. The two-fold symmetry related central copper(II) units along the *meso*-helical chain are shown in different colors for clarity.

**Figure 2:**
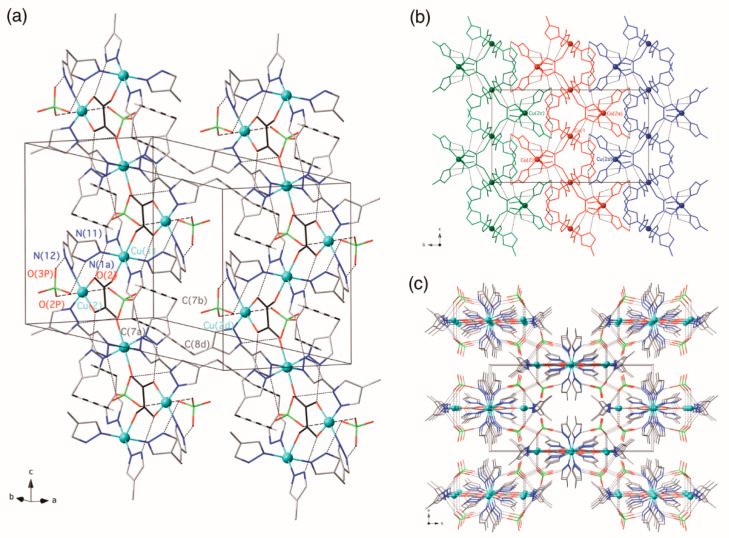
(**a**) View of the unit cell of **1** with the atom-numbering scheme of selected atoms [symmetry code: (a) 1 − *x*, − *y*, − *z*; (b) 1 − *x*, *y*, 1/2 − *z*; (d) 3/2 − *x*, 1/2 − *y*, − *z*]. Selected hydrogen bond distances (Å): N(11)–H(11)···O(2) = 2.807(2) Å, N(12)–H(12)···O(3P) = 2.951(3), and N(1)–H(1)···O(2Pc) = 2.957(3) (data from Table S2). The C–C and C–O bonds from oxalate are depicted by black sticks to follow the chain development. The intrachain N–H···O hydrogen bonds between the pyrazole and the oxalate ligands or the perchlorate anions are depicted by black dotted lines, while the intra- and interchain C–H···π type interactions between the neighboring pyrazole ligands are depicted by thick black and gray dashed lines, respectively; (**b**) projection view of the stacking of the cationic copper(II) chains along the crystallographic *a* axis [symmetry code: (a) 1 − *x*, − *y*, − *z*; (c) 1/2 − *x*, 1/2 − *y*, − *z*; (d) 3/2 − *x*, 1/2 − *y*, − *z*]. The adjacent layers are shown in different colors for clarity. Shortest intra- and interchain separations (Å): Cu(1)···Cu(2) = 5.9493(3), Cu(1)···Cu(1b) = 6.8420(1), Cu(2)···Cu(2c) = 8.9641(2), Cu(1)···Cu(2c) = 9.4934(3), Cu(2)···Cu(2d) = 9.7651(2), and Cu(1)···Cu(2d) = 9.8806(3); (**c**) perspective view of the crystal packing along the crystallographic *c* axis, showing the hydrogen-bonded supramoleculr 3D network of cationic copper(II) chains. The intrachain N–H···O and interchain C–H···O interactions between the pyrazole and the oxalate ligands or the perchlorate anions are indicated by black and gray dotted lines, respectively.

The crystal structure of 1 consists of oxalato-centered cationic copper(II) chains showing a regular alternation of mononuclear bis(pyrazole)oxalatocopper(II) and tetrakis(pyrazole)copper(II) units, [Cu(4-Hmpz)_2_(ox)] and [Cu(4-Hmpz)_4_]^2+^ respectively, together with weakly coordinated perchlorate anions (Figure 1 and Figure 2).

The two crystallographically independent Cu(1) and Cu(2) atoms from the [Cu(4-Hmpz)_4_]^2+^ and [Cu(4-Hmpz)_2_(ox)] fragments of **1** show a six-coordinate (4+2) axially elongated octahedral geometry (Figure 1a). The CuN_4_O_2_ chromophore at the Cu(1) atom, located on the crystallographic inversion center, is formed by four pyrazole-nitrogen atoms [N(2), N(2a), N(21), and N(21a)] in the equatorial plane and two oxalate-oxygen atoms [O(1) and O(1a)] from two neighboring [Cu(4-Hmpz)_2_(ox)] units in the axial positions [symmetry code: (a) 1 − *x*, − *y*, − *z*]. The CuN_2_O_4_ chromophore at the Cu(2) atom, located on the crystallographic two-fold symmetry axis, is formed by two pyrazole-nitrogen atoms from two pyrazole groups [N(22) and N(22b)] and two oxalate-oxygen atoms [O(2) and O(2b)] in the equatorial plane, the two axial positions being filled by two weakly coordinated perchlorate anions [O(1P) and O(1Pb)] [symmetry code: (b) 1 − *x*, *y*, 1/2 − *z*].

The oxalato centroligand from the [Cu(4-Hmpz)_2_(ox)] units of **1** adopts a four-connected µ*_3_-κ*^2^*O*^1^,*O*^2^*:κO*^2′^*:κO*^1′^ coordination mode (**III.3** in Scheme 1), with two short (*R* ≈ 1.96 Å) and two long (*R’* ≈ 2.38 Å) Cu–O distances. This situation contrasts with those found for the related neutral mononuclear and chain compounds of formulas [Cu(3,5-Hdmpz)_2_(ox-*κ*^2^*O*^1^,*O*^2^)(H_2_O)] (**3**) and [Cu(3-Hmpz)_2_(µ-ox*-κ*^2^*O*^1^,*O*^2^*:κO*^1′^)]_n_ (**4**) (3,5-Hdmpz = 3,5-dimethyl-1*H*-pyrazole and 3-Hmpz = 3-methyl-1*H*-pyrazole) [58,59]. In particular, the oxalate ligand acts as terminal side-on bidentate ligand (*κ*^2^*O*^1^,*O*^2^, see **I.2** in Scheme 1) in **3**, while adopting a three-connected bidentate/monodentate (µ*-κ*^2^*O*^1^,*O*^2^*:κO*^1′^, **II.5** in Scheme 1) coordination mode in **4** with two short equatorial [*R* = 1.950(2) Å] and one long axial [*R’* = 2.416(2) Å] Cu–O distances. Moreover, **1** exhibits a smaller asymmetry of the metal-to-oxalato bonds compared to **4**, as reflected by the values of the difference between the long and short Cu–O distances [Δ*R* = *R*^’^ − *R* = 0.42 (**1**) and 0.47 Å (**4**)].

For symmetry reasons, the mean equatorial planes at the alternating copper atoms along the chain are almost orthogonal for both **1** and **4** [*ψ* = 85.7° (**1**) and 81.5° (**4**)], being almost coplanar with respect to the mean oxalate plane [*ϕ* = 6.09° (**1**) and 5.8° (**4**)]. The values of the in-plane Cu–O–C bond angle are *α* = 147.7 (**1**) and 177.0° (**4**), whereas those of the Cu–O–C–O torsion angle are *β* = 21.9 (**1**) and 20.2° (**4**) [59]. Overall, this asymmetric bidentate/bis(monodentate) coordination mode of oxalato for **1** gives rise to a unique inverse triangular-based oxalato-centered copper(II) chain of branch-type topology featuring vertex-shared triangular copper(II) entities (Figure 1b,c). The two-fold symmetry-related Cu(1) and Cu(1b) atoms within each alternating chain of **1**, stand on the central chain axis while the centrosymmetrically-related Cu(2) and Cu(2a) atoms are alternatively located in opposite peripheral branches along the chain (Figure 1b,c). The mean equatorial planes of the two-fold symmetry-related propeller-like [Cu(4-Hmpz)_4_]^2+^ units are neither orthogonal with respect to the chain axis (*ρ* = 56.35°) nor parallel to each other (*θ* = 67.3°) (Figure 1b). Moreover, their principal N–Cu–N axes are rotated along the chain axis (*τ* = 27.0 and 46.4°) (Figure 1c). The regular alternation of left and right rotations along the chain leads to an overall achiral (*meso*-helical) chain. Hence, **1** constitutes a rare example of an oxalato-centered inverse copper(II) *meso*-helix [60].

In the crystal lattice of **1**, the *meso*-helical copper(II) chains running in the [001] direction are parallel stacked leading to segregated layer arrays of cationic chains within the crystallographic *ac* plane (Figure 2). Within each *meso*-helical chain, the pyrazole ligands establish moderate to weak hydrogen bonds with the carboxylate oxygen atoms from the oxalate centroligands and the weakly coordinated perchlorate anions (Figure 2a). The adjacent chains interact through additional very weak contacts involving the methyne and methyl groups from pyrazole ligands and the weakly coordinated perchlorate anions, leading to a supramolecular 3D network of *meso*-helical chains (Figure 2c). There are also moderate to weak, intra- and interchain C–H···π type interactions between the methyl-pyrazole groups from the [Cu(4-Hmpz)_4_]^2+^ and [Cu(4-Hmpz)_2_(ox)] units, respectively, and the pyrazole rings from the [Cu(4-Hmpz)_4_]^2+^ fragments (Figure 2a,b), as revealed by the values of the intra- and interchain distance between the methyl carbon atoms [C(7) and C(8)] and the centroid of the pyrazole rings from neighboring units (*h* = 3.651 and 3.976 Å, respectively).

#### 3.2.2. {[Cu(3,4,5-Htmpz)_4_]_2_[Cu(3,4,5-Htmpz)_2_(µ_3_-ox-*κ*^2^*O*^1^,*O*^2^:*κO*^2′^:*κO*^1′^)(H_2_O)(ClO_4_)]_2_ [Cu_2_(3,4,5-Htmpz)_4_(µ-ox-*κO*^1^,*O*^2^:*κ*^2^*O*^2′^,*O*^1′^)]}(ClO_4_)_4_·6H_2_O (**2**)

**Figure 3:**
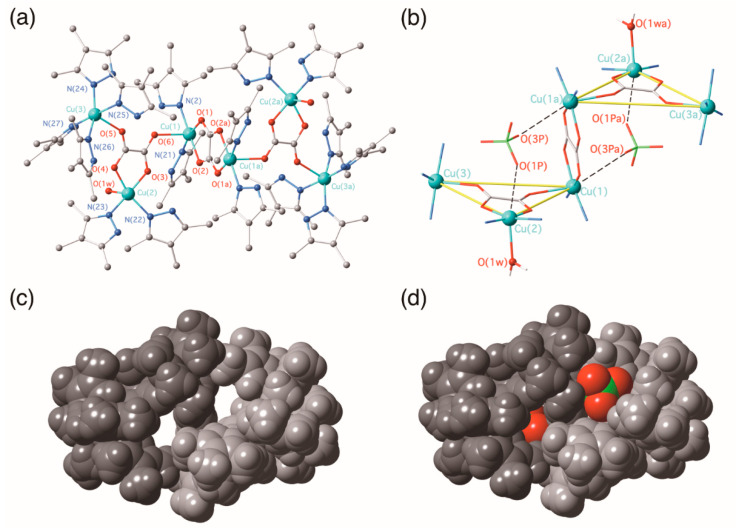
(**a**) Perspective view of the cationic hexacopper(II) complex of **2** with the atom numbering of the donor set at each copper site [symmetry code: (a) − *x*, 2 − *y*, − *z*]. Hydrogen atoms are omitted for clarity. Selected bond lengths (Å): Cu(1)–N(2) = 1.988(3), Cu(1)–N(21) = 1.975(2), Cu(2)–N(22) = 1.996(3), Cu(2)–N(23) = 1.963(3), Cu(3)–N(24) = 1.997(3), Cu(3)–N(25) = 2.030(3), Cu(3)–N(26) = 1.984(3), Cu(3)–N(27) = 2.004(3), Cu(1)–O(1) = 1.994(2), Cu(1)–O(2) = 2.015(2), Cu(2)–O(3) = 1.948(2), Cu(2)–O(4) = 1.994(2), Cu(1)–O(6) = 2.341(2), Cu(3)–O(5) = 2.256(2), Cu(2)–O(1w) = 2.432(3), Cu(2)–O(1P) = 2.851(11), and Cu(1)–O(3Pa) = 2.808(7) (data from Table S3); (**b**) projection view of the inverse oxalato-centered hexacopper(II) skeleton of branch-type topology with the atom numbering scheme of selected atoms, showing the two perchlorate anions acting as additional linkers. The weak axial coordinative bonds with the perchlorate anions are depicted by dashed lines. The intramolecular copper-copper separations through the µ_3_-ox-*κ*^2^*O*^1^,*O*^2^:*κO*^2′^:*κO*^1′^ centroligand within the two triangular copper(II) entities are evidenced by thick yellow solid lines; (**c**,**d**) space-filling model representation of the *S*-shaped hexacopper(II) capsule including and excluding the two encapsulated perchlorate anions, respectively. The two centrosymmetrically-related triangular copper(II) entities are depicted in dark and pale gray colors for clarity.

**Figure 4:**
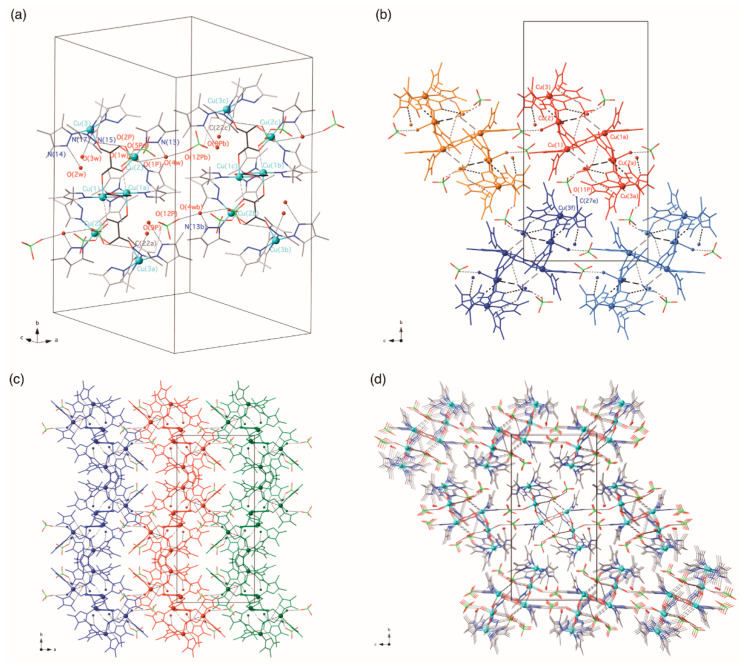
(**a**) View of the unit cell of **2** with the atom numbering of selected atoms [symmetry code: (a) − *x*, 2 − *y*, − *z*; (b) 1 − *x*, 2 − *y*, − *z*; (c) 1 + *x*, *y*, *z*; (d) 1 − *x*, 2 − *y*, 1 − *z*]. Selected hydrogen bond distances (Å): N(15)–H(15)···O(2P) = 3.065(5), N(17)–H(17)···O(3w) = 2.600(9), N(13)–H(13)···O(4w) = 2.785(5), N(14)–H(14)···O(2w) = 2.828(4), N(11)–H(11)···O(3) = 2.809(3), N(16)–H(16)···O(4) = 2.863(3), N(12)–H(12)···O(2) = 3.345(3), O(2w)···O(6) = 2.893(4), O(1w)···O(5Pd) = 3.008(8), O(4w)···O(12Pb) = 2.921(16), O(4w)···O(1P) = 3.124(15), O(3w)···O(8P) = 3.216(9), C(6)–H(6C)···O(1) = 3.100(5), C(27e)–H(27e)···O(11P) = 3.36(2), and C(22a)–H(22a)···O(9P) = 3.467(8) (data from Table S4). The C–C and C–O bonds from oxalate are depicted by black sticks to follow the hexanuclear development. The N–H···Ow, N–H···O, and Ow–H···O hydrogen bonds involving the pyrazole ligands, the perchlorate anions, and the water molecules of crystallization are depicted by black dotted lines, while the C–H···O type contacts involving the pyrazole ligands and the perchlorate anions are depicted by gray dotted lines; (**b**) projection views of neighboring hexanuclear entities along the crystallographic *a* axis with the atom numbering of selected atoms [symmetry code: (e) 1 − *x*, 1/2 + *y*, 1/2 − *z*; (f) − *x*, 1/2 + *y*, 1/2 − *z*]. The adjacent hexanuclear entities are shown in different colors for clarity. Shortest intra- and intermolecular separations (Å): Cu(1)···Cu(2) = 5.419(1), Cu(2)···Cu(3) = 5.703(1), Cu(1)···Cu(3) = 7.140(1), Cu(1)···Cu(1a) = 5.225(1), Cu(1)···Cu(3f) = 8.846(1), Cu(3a)···Cu(3f) = 9.384(1), Cu(3a)···Cu(2b) = 11.430(1), and Cu(2)···Cu(2b) = 11.616(1); (**c**) projection view of the layer stacking of hexanuclear entities along the crystallographic *c* axis. The adjacent layers are shown in different colors for clarity; (**d**) perspective view of the crystal packing along the crystallographic *a* axis.

The structure of **2** contains cationic centrosymmetric oxalato-centered inverse hexanuclear entities made up of an inner dinuclear [Cu_2_(3,4,5-Hdmpz)_4_(µ-ox)]^2+^ motif and two outer mononuclear [Cu(3,4,5-Htmpz)_4_]^2+^ units which are interconnected through two mononuclear [Cu(3,4,5-Htmpz)_2_(µ_3_-ox)(H_2_O)] units. Weakly coordinated and free perchlorate anions and crystallization water molecules are also present (Figure 3 and Figure 4).

The two pairs of centrosymmetrically-related Cu(1)/Cu(1a) and Cu(2)/Cu(2a) atoms from the inner [Cu_2_(3,4,5-Hdmpz)_4_(µ-ox)]^2+^ and the two intermediate [Cu(3,4,5-Htmpz)_2_(H_2_O)(µ_3_-ox)] fragments exhibit a six-coordinate (4 + 1 + 1) axially elongated octahedral geometry (Figure 3b). The equatorial plane of each CuN_2_O_4_ chromophore at the Cu(1) and Cu(2) atoms is built by two pyrazole-nitrogen atoms [N(2) and N(21) at Cu(1) and N(22) and N(23) at Cu(2)] and two oxalate-oxygen atoms [O(1) and O(2) at Cu(1) and O(3) and O(4) at Cu(2)]. One of the two axial positions is occupied by a water molecule [O(1w) at Cu(2)] and one oxalate-oxygen atom [O(6) at Cu(1)] of the intermediate [Cu(3,4,5-Htmpz)_2_)(H_2_O)(µ_3_-ox] unit, the remaining axial positions being occupied by a weakly coordinated perchlorate anion acting as an additional linker between the Cu(1) and Cu(2a) atoms. The centrosymmetrically-related Cu(3)/Cu(3a) atoms from the two outer [Cu(3,4,5-Htmpz)_4_]^2+^ fragments exhibit a five-coordinate (4 + 1) apically elongated square pyramidal geometry. The CuN_4_O chromophore at the Cu(3) atom is formed by four pyrazole-nitrogen atoms [N(24), N(25), N(26), and N(27)] in the basal plane and one oxalate-oxygen atom [O(5)] of the intermediate [Cu(3,4,5-Htmpz)_2_(H_2_O)(µ_3_-ox)] unit in the apical position (Figure 3a).

The oxalato centroligands from the inner dicopper(II) fragments and the two intermediate copper(II) units of **2** act as either symmetric or asymmetric inverse coordination centers. They adopt µ*-κ*^2^*O*^1^, *O*^2^*:κ*^2^*O*^2′^,*O*^1′^ (**II-6**) or µ_3_*-κ*^2^*O*^1^,*O*^2^*:κO*^2′^*:κO*^1′^ (**III-3**) coordination modes, with four short [*R* ≈ 1.99 and 2.02 Å] or two short [*R* ≈ 1.95 and 1.99 Å] plus two long [*R’* ≈ 2.26 and 2.34 Å] copper-to-oxygen distances, respectively. However, the mean equatorial planes at each copper atom are almost coplanar with respect to the mean oxalate plane for both the inner [Cu_2_(3,4,5-Hdmpz)_4_(µ-ox)]^2+^ and outer [Cu(3,4,5-Htmpz)_4_]^2+^ fragments (*ϕ* = 5.4 and 13.9°, respectively). The symmetric bis(bidentate) coordination mode within the inner [Cu_2_(3,4,5-Hdmpz)_4_(µ-ox)]^2+^ motif is rather common in polynuclear oxalato complexes as found earlier for the related pair of cationic dicopper(II) complexes of general formula [Cu_2_L_6_(µ*-*ox*-κ*^2^*O*^1^,*O*^2^*:κ*^2^*O*^2′^,*O*^1′^)](ClO_4_)_2_·2H_2_O [L = 3,5-Hdmpz (**5a**) and 3,4,5-Htmpz (**5b**)] [50]. However, **5a** and **5b** show a significant asymmetry of the metal-to-oxalato bonds [*R* = 1.98 (**5a**)/1.97 Å (**5b**) and *R’* = 2.12 (**5a**)/2.11 Å (**5b**), with Δ*R* = *R*^’^ − *R* = 0.15 (**5a**)/0.14 Å (**5b**)] resulting from the five-coordinate trigonal bipyramidal geometry [50]. For symmetry reasons, the mean equatorial planes at each copper atom within the central dinuclear motif of **2** are parallel (*ψ* = 0°). The asymmetric bidentate/bis(monodentate) coordination mode of the oxalate center (**III-3**) within the two intermediate [Cu(3,4,5-Htmpz)_2_(H_2_O)(µ_3_-ox)] fragments of **2** is similar to that found in the [Cu(4-Hmpz)_2_(µ_3_-ox)] unit of **1**. Like in **1**, the intermediate fragments adopt an in-plane perpendicular planar conformation with respect to both the central [Cu_2_(3,4,5-Hdmpz)_4_(µ-ox)]^2+^ (*ψ* = 77.5° with *α* = 126.5° and *β* = 19.0°) and peripheral [Cu(3,4,5-Htmpz)_4_]^2+^ fragments (*ψ* = 87.8° with *α* = 138.1° and *β* = 17.7°), the mean equatorial planes of the copper atoms being almost coplanar with respect to the mean oxalato plane (*ϕ* = 5.4 and 13.9°). However, the asymmetry of the metal-oxalato bonds is greater in **1** [Δ*R* = *R*^’^ − *R* = 0.42 Å] than in **2** [Δ*R* = *R*^’^ − *R* = 0.26 and 0.33 Å].

Overall, these structural features give rise to a unique inverse triangular-based oxalato-centered hexacopper(II) skeleton of branch-type topology featuring two parallel planar triangular copper(II) entities (Figure 3b). Interestingly, the hexacopper(II) entity possesses a *S*-shaped conformation with two void spaces that are filled by the two weakly coordinated perchlorate anions, giving rise to an oblate spheroid-like shape capsule (Figure 3c,d). Hence, **2** constitutes a rare example of anionic guest encapsulation by an oxalato-centered inverse polynuclear copper(II) complex in the solid-state [61,62].

In the crystal lattice of **2**, the cationic hexacopper(II) units are well-separated from each other by the remaining non-coordinated perchlorate anions (Figure 4d). The hexanuclear entities are involved in a variety of moderately to relatively weak hydrogen bonds with both the weakly coordinated and free perchlorate anions and the water molecules of crystallization through their N–H pyrazole groups, the carbonyl oxygen atoms from oxalate, and the coordinated water molecules (Figure 4a). Moreover, there are moderately to relatively weak, intramolecular hydrogen bonds between the N–H pyrazole groups and the carbonyl-oxygen atoms from oxalato and intermolecular hydrogen bonds between the perchlorate anions and the water molecules of crystallization. Very weak intramolecular contacts between the methyl groups from pyrazole ligands and the carbonyl-oxygen atoms from oxalato and intermolecular contacts between the hydrogen-bonded perchlorate anions and the methyl groups from pyrazoles of adjacent {Cu^II^_6_} entities also occur (Figure 4a,b). They give rise to a dense-packed multilayer supramolecular structure made up of corrugated layers growing in the crystallographic *bc* plane that are parallel stacked along the crystallographic *a* axis (Figure 4b,c).

### 3.3. Magnetic Properties

**Figure 5:**
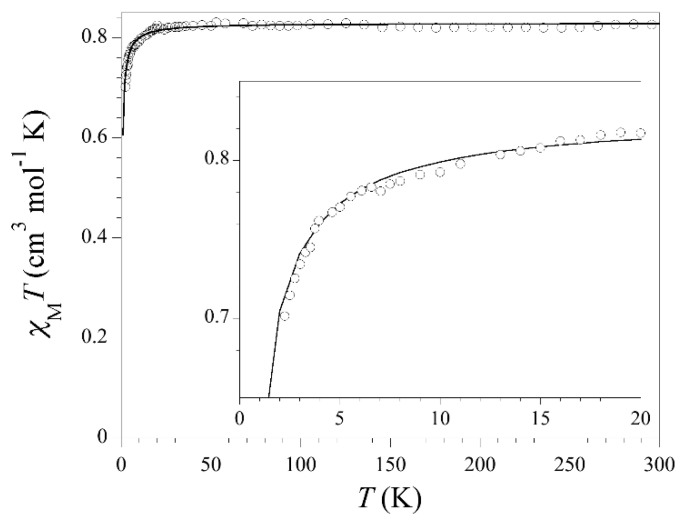
Temperature dependence of *χ*_M_*T* for **1** (◯). The inset shows a detail of the low-temperature region. The solid lines correspond to the best-fit curves through Equation (1) (see text).

**Figure 6:**
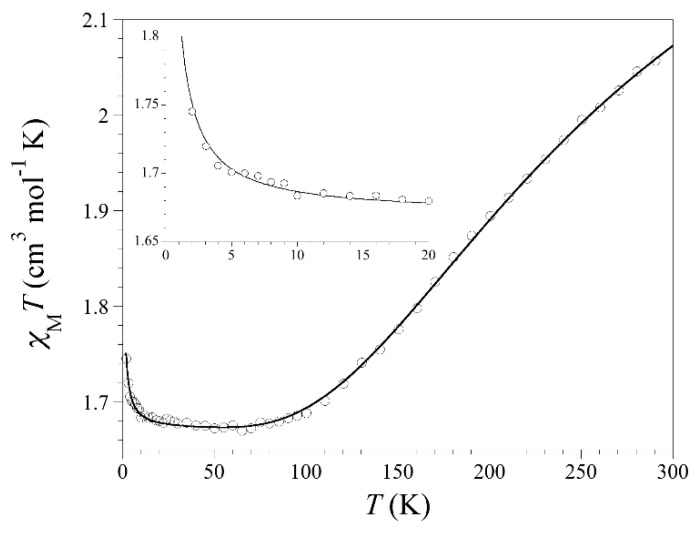
Temperature dependence of *χ*_M_*T* for **2** (◯). The inset shows a detail of the low-temperature domain. The solid line corresponds to the best-fit curve through Equation (2) (see text).

Figure 5 and Figure 6 show the magnetic properties of **1** and **2** in the form of the *χ*_M_*T versus T* plots (*χ*_M_ being the dc molar magnetic susceptibility per formula unit and *T* the absolute temperature).

**1** exhibits a magnetic behavior which is typical of very weak antiferromagnetically coupled copper(II) ions (Figure 5). At room temperature, *χ*_M_*T* for **1** is equal to 0.83 cm^3^ mol^−1^ K, a value which is as expected for two magnetically isolated copper(II) ions [*χ*_M_*T* = 2 × (*Nβ*^2^*g*_Cu_^2^/3*k*_B_)*S*_Cu_(*S*_Cu_ + 1) = 0.83 cm^3^ mol^−1^ K with *g*_Cu_ = 2.1 and *S*_Cu_ = 1/2]. Upon cooling, *χ*_M_*T* remains constant down to 20 K, and it further decreases to 0.70 cm^3^ mol^−1^ K at 2.0 K (inset of Figure 5). This small negative deviation from the Curie law in the low-temperature region is most likely due to very weak intrachain antiferromagnetic interactions between the copper(II) ions across the µ_3_-ox-*κ*^2^*O*^1^,*O*^2^:*κO*^2′^:*κO*^1′^ centroligand (see Figure 1b).

Having in mind these considerations, the analysis of the magnetic susceptibility data of **1** was carried out by means of the Bonner–Fisher expression for a linear regular copper(II) chain [Equation (1) with *x* = |*J*|/*k*_B_*T*] [63]:

*χ*_M_*T* = (2*Nβ*^2^*g*^2^/*k*_B_)(0.25 + 0.074975*x* + 0.075235*x*^2^)/(1.0 + 0.9931*x* + 0.172135*x*^2^ + 0.757825*x*^3^) (1)

**Scheme 2:**
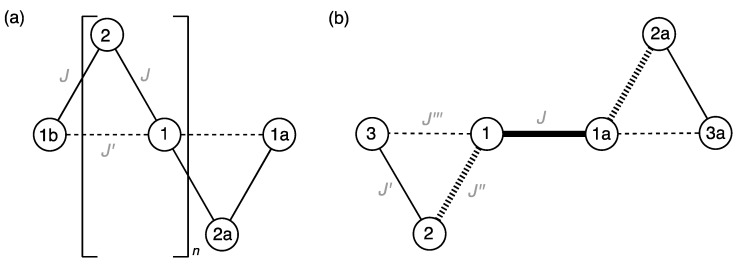
Magnetic coupling model for **1** (**a**) and **2** (**b**).

In this expression, *J* is the magnetic coupling parameter between the Cu(1) and Cu(2) atoms (Scheme 2a), the spin Hamiltonian being defined as ***H*** = −*J∑**S***_1,i_ · ***S***_2,i**+1**_ (*i* running from 1 to *n* − 1), and *g* is the average Landé factor of each copper(II) ion (*g* = *g*_1_ = *g*_2_ = *g*_Cu_). In this case, the intrachain magnetic coupling between the Cu(1) and Cu(1b) atoms across the long/long axial-axial pathway of the µ_3_-ox-*κ*^2^*O*^1^,*O*^2^:*κO*^2′^:*κO*^1′^ centroligand (*J’*) is negligible compared to that between the one concerning Cu(1) and Cu(2) atoms through the long/short axial-equatorial pathway (*J*) (Scheme 2a). Least-squares best-fit parameters are *J* = −0.42(1) cm^−1^, *g* = 2.105(2), and *R* = 2.0 × 10^−5^ (*R* is the agreement factor defined as *R* = ∑[(*χ*_M_*T*)_exp_ − (*χ*_M_*T*)_calcd_]^2^/∑[(*χ*_M_*T*)_exp_]^2^). The theoretical curve matches very well the experimental one (solid line in Figure 5). In particular, it nicely reproduces the observed small decrease of *χ*_M_*T* at very low temperatures (solid line in the inset of Figure 5).

The magnetic behavior of **2** is characteristic of the coexistence of strong antiferro- (high temperature region) and very weak ferromagnetic (low temperature region) interactions within the hexacopper(II) unit (Figure 6). The value of *χ*_M_*T* of 2.07 cm^3^ mol^−1^ K at room temperature is rather lower than that expected for six magnetically non-interacting copper(II) ions [*χ*_M_*T* = 6 × (*Nβ*^2^*g*_Cu_^2^/3*k*_B_)*S*_Cu_(*S*_Cu_ + 1) = 2.49 cm^3^ mol^−1^ K with *g*_Cu_ = 2.1 and *S*_Cu_ = 1/2]. *χ*_M_*T* continuously decreases upon cooling reaching a sort of plateau in the temperature range 100 to ca. 20 K with a value of *χ*_M_*T* equal to 1.68 cm^3^ mol^−1^ K. This value is very close to the expected one for four magnetically isolated copper(II) ions [*χ*_M_*T* = 6 × (*Nβ*^2^*g*_Cu_^2^/3*k*_B_)*S*_Cu_(*S*_Cu_ + 1) = 1.66 cm^3^ mol^−1^ K with *g*_Cu_ = 2.1 and *S*_Cu_ = 1/2]. Both features unambiguously support the occurrence of a diamagnetic singlet (*S* = 0) spin ground state for the inner dicopper(II) unit due to the expected strong intramolecular antiferromagnetic interaction between the copper(II) ions through the µ*-*ox-*κ*^2^*O*^1^,*O*^2^*:κ*^2^*O*^2′^,*O*^1′^ centroligand (see Figure 3b). Below 20 K, *χ*_M_*T* slightly increases to attain a value of 1.75 cm^3^ mol^−1^ K at 2.0 K (inset of Figure 6). This small increase of *χ*_M_*T* at very low temperatures is most likely due to a very weak intramolecular ferromagnetic interaction between the copper(II) ions through the µ_3_-ox-*κ*^2^*O*^1^,*O*^2^:*κO*^2′^:*κO*^1′^ centroligand (see Figure 3b).

In a first attempt, the magnetic susceptibility data of **2** were accordingly analyzed by the appropriate expression for the sum of magnetically non-interacting inner [Cu(1)/Cu(1a)] and outer [Cu(2)/Cu(3) and Cu(2a)/Cu(3a)] dinuclear fragments derived through the Bleaney–Bowers expression [Equation (2)] [64]:

*χ*_M_*T* = (2*Nβ*^2^*g*^2^/*k*_B_)/[3 + exp(–*J*/*kT*)] + (4*Nβ*^2^*g*^2^/*k*_B_)/[3 + exp(−*J’*/*kT*)] (2)

In this expression, *J* and *J’* are the magnetic coupling parameters between the inner Cu(1) and Cu(1a) atoms and between the intermediate Cu(2)/Cu(2a) and the outer Cu(3)/Cu(3a) atoms, respectively (Scheme 2b), the corresponding spin Hamiltonian being defined as ***H*** = −*J**S*****_1_** · S**_1′_** − *J’*(***S*_2_** · ***S*_3_** + ***S*_2′_** · **S_3′_**) (see Figure 3b), and *g* is the average Landé factor of the six copper(II) ions (*g* = *g*_1_ = *g*_2_ = *g*_3_ = *g*_Cu_). The least-squares fit of the experimental data led to the following set of parameters: *J* = −348(2) cm^−1^, *J’* = +0.28(2) cm^−1^, and *g* = 2.110(1) with *R* = 3.0 × 10^−5^. The calculated curve (solid line in Figure 6) reproduces quite well the experimental data in the whole temperature range explored.

In a second attempt, the magnetic susceptibility data of **2** were analyzed by matrix diagonalization of the spin Hamiltonian for a linear hexanuclear copper(II) complex, ***H*** = −*J**S*****_1_** · ***S*_1′_** − *J’*(***S*_2_** · ***S*_3_** + ***S*_2′_** · ***S*_3′_**) − *J’’*(***S*_1_** · ***S*_2_** + ***S*_1′_** · ***S*_2′_**), where *J’’* is the intramolecular magnetic coupling parameter between the inner Cu(1) and intermediate Cu(2) atoms (Scheme 2b). In this case, the intramolecular magnetic coupling between the inner Cu(1) and outer Cu(3) atoms across the long/long axial-axial pathway of the µ_3_-ox-*κ*^2^*O*^1^,*O*^2^:*κO*^2′^:*κO*^1′^ centroligand (*J’’’*) is negligible compared to those between the intermediate Cu(2) and outer Cu(3) atoms (*J’*) and between the inner Cu(1) and intermediate Cu(2) atoms (*J’’*) across the long/short axial-equatorial pathway (Scheme 2b). The least-squares fit of the experimental data gave *J* = −348(12) cm^−1^, *J’* = +0.28(3) cm^−1^, and *J’’* = 0(30) cm^−1^ with *g* = 2.110(2) and *R* = 1.6 × 10^−5^ [65]. The calculated *J* and *J’* values are identical to those obtained previously, while the *J’’* value is practically null, as expected from our preliminary simulations. However, the error associated to the *J’’* value is so huge that it remains undetermined.

### 3.4. Magneto-Structural Correlations

**Scheme 3:**
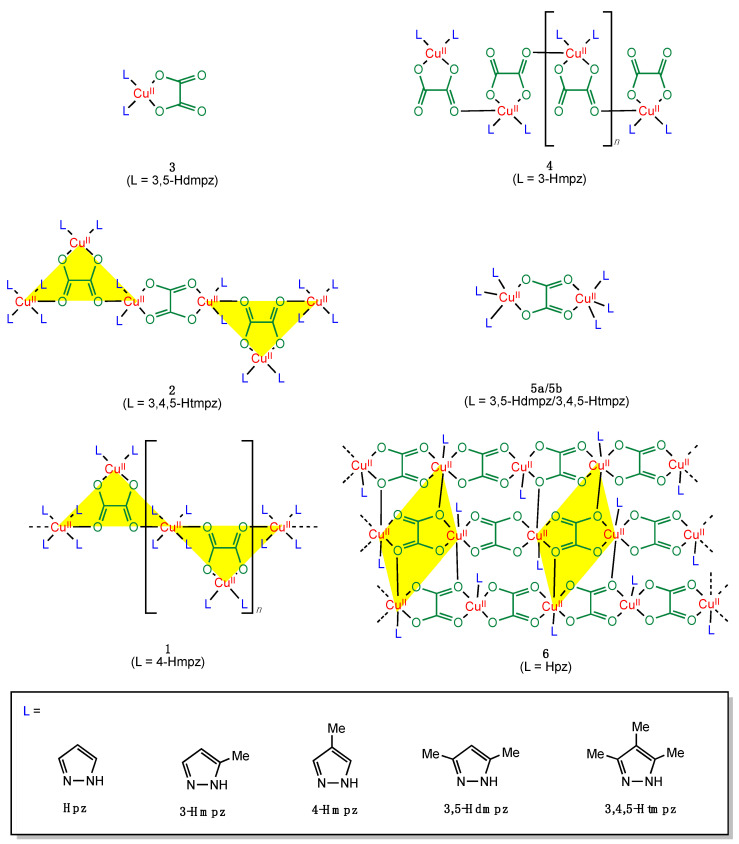
Chemical structures of the inverse copper(II) compounds with oxalato as centroligand and pyrazole and its polymethyl-substituted derivatives as terminal ligands: triangular-based branch-type chain and hexanuclear compounds (**1** and **2**), asymmetric zig-zag chains (**4**), symmetric dinuclear complexes (**5a**/**5b**), and rectangular-based alternating sheet-like compounds (**6**). The Werner-type mononuclear complex (**3**) has been included for completeness.

In order to account for the unique structural features and magnetic properties of **1** and **2**, we will refer to related examples of inverse polynuclear copper(II) complexes with oxalato as inverse coordination center and pyrazole or its polymethyl-substituted derivatives as terminal ligands (Scheme 3). Selected magneto-structural data for the broad oxalato-centered inverse copper(II)-pyrazole family are shown in Table 2.

The strong antiferromagnetic coupling found in **2** (*J* = −348 cm^−1^) agrees with that previously observed for the related neutral copper(II) 2D coordination polymer of formula {[Cu(Hpz)(µ_4_-ox-*κO*^1^:*κ*^2^*O*^1^,*O*^2^:*κO*^2′^:*κ*^2^*O*^2′^,*O*^1′^)][Cu(Hpz)(µ-ox-*κ*^2^*O*^1^,*O*^2^:*κ*^2^*O*^2′^,*O*^1′^)]}*_n_* (**6**) with 1*H*-pyrazole (Hpz) as terminal ligand (*J* = −312 cm^−1^) (see Scheme 3) [59]. In both cases, the unpaired electron at each copper(II) ion in a square pyramidal surrounding is delocalized in the d(x^2^-y^2^)-type magnetic orbital (the *x* and *y* axes being roughly defined by the short basal Cu–O bonds). A large spin delocalization is predicted on the µ-ox-*κ*^2^*O*^1^,*O*^2^:*κ*^2^*O*^2′^,*O*^1′^ centroligand, due to the almost coplanar disposition of the metal basal planes and the mean oxalato plane [*ϕ* = 5.4 (**2**) and 2.8° (**6**) with *ψ* = 0 (**2**) and 0° (**6**); Table 2] [59]. This situation contrasts with the intermediate value of the antiferromagnetic coupling found for the related pair of cationic dicopper(II) complexes **5a** and **5b** (*J* = −129 and −161 cm^−1^, respectively) (Scheme 3) [50]. This change in the size of the magnetic coupling is explained by the decrease of the spin delocalization of the unpaired electron from the trigonal bipyramidal copper(II) ions of **5a** and **5b** on the µ-ox-*κ*^2^*O*^1^,*O*^2^:*κ*^2^*O*^2′^,*O*^1′^ centroligand, the magnetic orbital at each copper(II) ion being of the d(*z*^2^)-type (the *z* axis pointing toward one of the two Cu–O_ox_ bonds). Then, a poor spin density is expected on the equatorial positions [50].

**Scheme 4:**
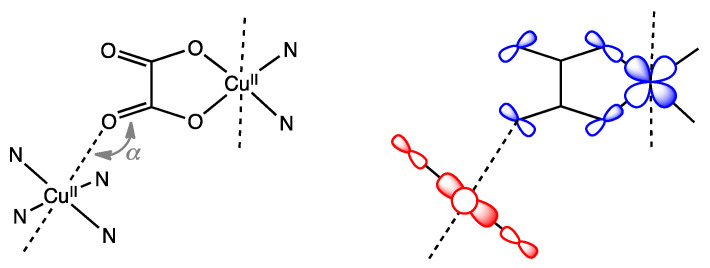
Illustration of the relative orientation of the d(x^2^ − y^2^)-type magnetic orbitals of the copper(II) ions for oxalato-centered dicopper(II) entities with a mutually perpendicular orientation of the metal equatorial planes. The solid and dashed lines represent short and long bonds, respectively.

**Figure 7:**
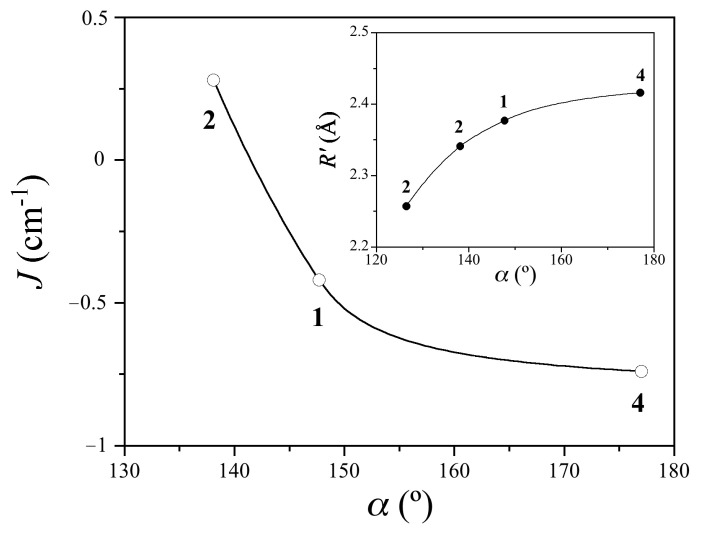
Variation of the magnetic coupling parameter (*J*) (○) and the long axial Cu–O distance (*R’*) (●) with the the Cu–O–C angle (*α*) for oxalato-centered inverse copper(II)-pyrazole compounds (data from Table 2). The solid lines are only eye-guides.

As far as the very weak ferro- or antiferromagnetic interactions found in **2** and **3** are concerned (*J* = +0.28 and −0.42 cm^−1^, respectively; Table 2), they are as expected for an in-plane perpendicular planar conformation of the equatorial planes at the metal atoms [*β* = 17.7 (**2**) and 21.9° (**3**) with *ψ* = 87.8 (**2**) and 85.7° (**3**); Table 2]. In this case, the d(x^2^−y^2^)-type magnetic orbitals of the axially elongated octahedral copper(II) ions are perpendicular to each other in two different planes leading to a poor *δ*-type orbital overlap along the long apical position through the µ_3_-ox-*κ*^2^*O*^1^,*O*^2^:*κO*^2′^:*κO*^1′^ pathway (Scheme 4). A similar situation applies for the related weak antiferromagnetically coupled copper(II) chain **4** (*J* = −0.74 cm^−1^), where a quasi perpendicular conformation of the metal basal planes of the apically elongated square pyramidal copper(II) ions is achieved through the µ-ox-*κ*^2^*O*^1^,*O*^2^:*κO*^1′^ pathway (*β* = 20.2° and *ψ* = 81.5°; Table 2) (Scheme 3) [59]. In all these cases, the *δ*-type orbital overlap between the d(x^2^−y^2^)-type magnetic orbitals along the long apical position would mainly depend on the Cu–O–C bond angle (*α*). It seems that the orbital overlap would be strictly zero for *α* values close to that occurring in **2** (*α* = 138.1°; Table 2), so that the antiferromagnetic contribution, which is proportional to the square of the orbital overlap, becomes null and the ferromagnetic one dominates (example of accidental orthogonality) [40]. The orbital overlap would increase as the *α* value increases leading to a dominant antiferromagnetic contribution to the overall magnetic coupling for **1** and **4** [*J* = −0.42 (**1**) and −0.74 cm^−1^ (**4**) with *α* = 147.7 (**1**) and 177.0° (**4**); Table 2] (Figure 7). That being so, the ferromagnetic and antiferromagnetic contributions would cancel each other for a critical value of *α* close to 141.5° for which *J* = 0 (Figure 7). Interestingly, the values of *R’* increase continuously with the *α* values for this series of copper(II) complexes with an in-plane perpendicular planar conformation (inset of Figure 7). Indeed, the orbital overlap would also decrease with increasing the axial Cu–O distance and, consequently, the antiferromagnetic contribution to the overall magnetic coupling. However, this is the opposite trend to that found for **1**, **2**, and **4** (*J* = +0.28 (**2**), −0.42 (**1**) and −0.74 cm^−1^ (**4**) with *R’* = 2.341 (**2**), 2.377 (**1**) and 2.416° (**4**); Table 2). This expected minor but non-negligible distance dependence of the antiferromagnetic contribution likely explains the observed non-linear angular dependence of the magnetic coupling along this series.

## 4. Conclusions

In this work, we investigated the influence of the ligand effects on the synthesis, crystal structure, and magnetic properties of a related pair of inverse copper(II) complexes (**1** and **2**) with oxalato as centroligand and 4-methyl- (L = 4-Hmpz) or 3,4,5-trimethyl-substituted (L = 3,4,5-Htmpz) pyrazoles as terminal ligands. Hence, **1** and **2** are two unique examples of a very weak antiferromagnetically coupled inverse coordination polymer (ICP) and strong to very weak antiferro/ferromagnetically coupled inverse polynuclear complex (IPC), featuring a four-connected oxalato-centered triangular-based chain and hexanuclear structures of branch-type topology. They were obtained by varying the Cu:pyrazole molar ratio during the inverse oxalato-mediated self-assembly process. Interestingly, **1** (L = 4-Hmpz) and **5a** (L = 3,5-Hdmpz) or **5b** (L = 3,4,5-Htmpz) constitute a special case of linkage isomers, as well as **3** (L = 3,5-Hdmpz) and **4** (L = 3-Hmpz), while **2** (L = 3,4,5-Htmpz) and **6** (L = Hpz) are unique on their own (see Scheme 3). It seems that the position and number of methyl substituents at the terminal pyrazole ligands are responsible for the distinct coordination mode and connectivity of the oxalato centroligand and eventually the different nuclearity and dimensionality [*n*D, with *n* = 0 (**2**, **3**, **5a**, and **5b**), 1 (**1** and **4**), or 2 (**6**)], as far as the synthetic reaction conditions, are identical for each pair of linkage isomers.

This study provides further insights into the ligand design, structural chemistry, and magnetochemistry of the well-known class of oxalato-centered inverse copper(II) IPCs and ICPs with pyrazole and their polymethyl-substituted derivatives possessing varying nuclearity and dimensionality. They range from traditional Werner-type mononuclear (**3**) to inverse dinuclear complexes (**5a** and **5b**) and chains (**4**), or from triangular-based hexanuclear complexes (**2**) and chains (**1**) to rectangular-based sheet-like polymers (**6**) with very weak to strong ferro- and/or antiferromagnetic couplings depending on the coordination mode of the oxalato centroligand. In this respect, new magneto-structural correlations have emerged for the series of oxalato-centered dicopper(II) complexes with an in-plane perpendicular planar conformation. This study completes and further extends the previous ones on the related series of symmetric and asymmetric oxalato-centered dicopper(II) complexes with a coplanar conformation, thus providing a unified vision on the magneto-structural correlations for this large class of oxalato-centered inverse copper(II)-pyrazole complexes.

## Figures and Tables

**Table 1:** Summary of crystal data and structure refinement for **1** and **2.**

Compound	1	2
CCDC number	2076622	2076621
Formula	C_26_H_36_Cl_2_Cu_2_N_12_O_12_	C_102_H_176_Cl_6_Cu_6_N_32_O_44_
Fw	906.65	3148.68
Crystal system	Monoclinic	Monoclinic
Space group	*C*2/*c*	*P*2_1_/*c*
*a*/Ǻ	12.3020(3)	15.3910(14)
*b*/Ǻ	23.0520(6)	30.496(3)
*c*/Ǻ	13.6840(2)	17.1782(17)
α/°	90	90
β/°	95.1130(13)	113.241(4)
γ/°	90	90
*V*/Ǻ^3^	3865.15(15)	7408.6(12)
*Z*	4	2
*D_c_*/g cm^−3^	1.558	1.411
*T*/K	293(2)	293(2)
*μ*/mm^−1^	1.310	1.039
*F*(000)	1856	3280
Refl. Collected	8741	116,008
Refl. indep. [*R*_int_]	4430 [0.0215]	15,028 [0.0789]
Refl. obs. [*I* > 2σ(*I*)]	10,171	10,171
Goodness-of-fit on *F*^2^	1.059	1.031
*R*_1_^a^ [*I* > 2σ(*I*)] (all)	0.0360 (0.0523)	0.0489 (0.0827)
*wR_2_*^b^ [*I* > 2σ(*I*)] (all)	0.0978 (0.1031)	0.1246 (0.1432)
Δρ_max, min_/e Å^−^^3^	0.485 and −0.433	0.653 and −0.566

^a^*R*_1_ = ∑||*F*_o_| − |*F*_c_||/∑|*F*_o_|. ^b^ *wR*_2_ = {∑*w*(*F*_o_^2^ − *F*_c_^2^)^2^/∑[*w*(*F*_o_^2^)^2^}^1/2^ and *w* = 1/[ σ ^2^(*F*_o_)^2^ + (*mP*)^2^ + *nP*] with *P* = (*F*_o_^2^ + 2*F*_c_^2^)/3, *m* = 0.0637 (**1**) and 0.0688 (**2**), and *n* = 0.0000 (**1**) and 4.1923 (**2**).

**Table 2:** Selected magneto-structural data for oxalato-centered inverse copper(II) complexes with pyrazole and polymethyl-substituted derivatives as peripheral ligands.

Compound ^a^	Coordination Mode	*R*^b^*/*Å	*R’*^c^*/*Å	*∆R*^d^/Å	*ϕ*^e^*/*°	*ψ*^f^*/*°	*α*^g^*/*°	*β*/°^h^	*r*^i^/Å	*J*^j^/cm^−1^	Ref.
**1**	µ_3_-*κ*^2^*O*^1^,*O*^2^:*κO*^2′^:*κO*^1′^	1.959	2.377	0.418	6.09	85.7	147.7	21.9	5.949	−0.42	This work
**2**	µ_3_*-κ*^2^*O*^1^,*O*^2^*:κO*^2′^*:κO*^1^ µ*-κ*^2^*O*^1^,*O*^2^*:κ*^2^*O^2′^*,*O*^1′^	1.948 1.994 1.994 2.016	2.257 2.341	0.309 0.347	13.9 5.4	77.5 87.8 0	126.5 138.1	19.0 17.7	5.418 5.703 5.224	n.a. +0.28 −348	This work
**4**	µ-*κ*^2^*O*^1^,*O*^2^:*κO*^1^	1.950	2.416	0.466	5.8	81.5	177.0	20.2	6.300	−0.74	[59]
**5a**	µ*-κ*^2^*O*^1^,*O*^2^*:κ*^2^*O*^2′^,*O*^1′^	1.976	2.122	0.146			114.9	2.0		−129	[50]
**5b**	µ*-κ*^2^*O*^1^,*O*^2^*:κ^2^O*^2′^,*O*^1′^	1.973	2.110	0.137			114.4	2.5		−161	[50]
**6**	µ_4_-*κO*^1^:*κ*^2^*O*^1^,*O*^2^:*κO*^2′^:*κ*^2^*O*^2′^,*O*^1′^	1.959	2.340	0.381	86.0	0	126.4	19.3	4.237 5.553	0 0	[59]
	µ-*κ*^2^*O*^1^,*O*^2^:*κ*^2^*O*^2′^,*O*^1′^	1.984 1.985			2.8	0			5.176	−312	

^a^ Compound notation: {[Cu(4-Hmpz)_4_][Cu(4-Hmpz)_2_(µ_3_-ox-*κ*^2^*O*^1^,*O*^2^:*κO*^2′^:*κO*^1′^)](ClO_4_)_2_}*_n_* (**1**); {[Cu(3,4,5-Htmpz)_4_]_2_[Cu(3,4,5-Htmpz)_2_(µ_3_-ox-*κ*^2^*O*^1^,*O*^2^:*κ*^2′^:*κO*^1′^)(H_2_O)(ClO_4_)]_2_[Cu_2_(3,4,5-Htmpz)_4_(µ-ox-*κ*^2^*O*^1^,*O*^2^:*κ*^2^*O*^2′^,*O*^1′^)]}(ClO_4_)_4_·6H_2_O (**2**); [Cu(3-Hmpz)_2_(µ-ox-*κ*^2^*O*^1^,*O*^2^:*κO*^1′^)]*_n_* (**4**); [Cu_2_(3,5-Hdmpz)_6_(µ*-*ox-*κ*^2^*O*^1^,*O*^2^*:κ^2^O*^2′^,*O*^1′^)](ClO_4_)_2_·2H_2_O (**5a**); [Cu_2_(3,4,5-Htmpz)_6_(µ-ox-*κ*^2^*O*^1^,*O*^2^*:κ*^2^*O*^2′^,*O*^1′^)](ClO_4_)_2_·2H_2_O (**5b**); {[Cu(Hpz)(µ_4_-ox-*κO*^1^:*κ*^2^*O*^1^,*O*^2^:*κO*^2′^:*κ*^2^*O*^2′^,*O*^1′^)][Cu(Hpz)(µ-ox-*κ*^2^*O*^1^,*O*^2^:*κ*^2^*O*^2′^,*O*^1′^)]}*_n_* (**6**) (see Scheme 3). ^b^ Average values of the equatorial Cu–O bond distances. ^c^ Values of the axial Cu–O interactions. ^d^ Values of the difference between the axial and equatorial Cu–O bond lengths (Δ*R* = *R*^’^ − *R*). ^e^ Values of the dihedral angle between the mean equatorial plane of the copper atom and the mean oxalato plane. ^f^ Values of the dihedral angle between the mean equatorial planes of the copper atoms. ^g^ Values of the axial Cu–O–C bond angle. ^h^ Values of the axial Cu–O–C–O torsion angle. ^i^ Values of the intramolecular copper-copper distance. ^j^ Values of the magnetic coupling between the copper(II) ions across the oxalate centroligand.

## Data Availability

The data are available by corresponding authors.

## Supplementary Materials

The following are available online, Figure S1: FT-IR spectra of **1** (a) and **2** (b), Figure S2: PXRD patterns of **1** (a) and **2** (b) compared to the calculated ones (bold lines), Tables S1 and S3: Selected bond distances (Å) and angles (°) for **1** and **2**, Tables S2 and S4: Selected hydrogen bond distances (Å) and angles (°) for **1** and **2**.

## Sample Availability

Samples of compounds **1** and **2** are available from the authors.
