# Supplementary material for: Ferro- and Antiferromagnetic Interactions in Oxalato-Centered Inverse Hexanuclear and Chain Copper(II) Complexes with Pyrazole Derivatives
Source: Molecules. 2021 May 10;26(9):2792. doi: 10.3390/molecules26092792 (PMC8126003; doi:10.3390/molecules26092792)
Supplement: Supplementary file 1 [file molecules-26-02792-s001.zip › molecules-1206776-SI.pdf]

Table S1. Selected bond distances (Å) and angles (°) for **1**[a]

Cu(1)-O(1)	2.3766(14)	Cu(2)-O(2)	1.9585(14)
Cu(1)-N(2)	2.011(2)	Cu(2)-N(22)	1.964(2)
Cu(1)-N(21)	2.045(2)	Cu(2)-O(1P)	2.496(4)
N(2)-Cu(1)-O(1)	88.28(6)	O(2)-Cu(2)-O(2b)	83.58(8)
N(2)-Cu(1)-N(21)	89.23(7)	O(2)-Cu(2)-N(22)	174.03(7)
N(2)-Cu(1)-N(21a)	90.77(7)	O(2)-Cu(2)-N(22b)	90.92(7)
N(2)-Cu(1)-N(2a)	180.0	N(22)-Cu(2)-N(22b)	94.67(11)
N(21)-Cu(1)-O(1)	93.26(6)	O(2)-Cu(2)-O(1P)	90.62(6)
N(2a)-Cu(1)-O(1)	91.72(6)	O(2b)-Cu(2)-O(1P)	89.67(6)
N(21a)-Cu(1)-O(1)	86.75(6)	N(22)-Cu(2)-O(1P)	91.65(7)
N(21)-Cu(1)-N(21a)	180.0	N(22b)-Cu(2)-O(1P)	88.08(7)
O(1a)-Cu(1)-O(1)	180.00(10)	O(1P)-Cu(2)-O(1Pb)	179.61(9)

[a]Symmetry code: (a) 1 – *x*, – *y*, – *z*; (b) 1 – *x*, *y*, 1/2 – *z*.

Table S2. Hydrogen bond distances (Å) and angles (°) in **1**[a,b,c]

D-H···A	d(D-H)	d(H···A)	d(D···A)	<(DHA)
N(1)-H(1)···O(1a)	0.86	2.53	3.082(3)	122.5
N(1)-H(1)···O(2Pc)	0.86	2.18	2.957(3)	149.4
N(11)-H(11)···O(2)	0.86	1.95	2.807(2)	172.1
N(12)-H(12)···O(3P)	0.86	2.13	2.951(3)	158.5
C(31)-H(31)···O(1a)	0.93	2.55	3.073(3)	116.1
C(52)-H(52)···O(4Pd)	0.93	2.58	3.503(3)	175.9
C(7)-H(7B)···O(1Pe)	0.96	2.90	3.600(3)	131.0

[a]D = H-donor and A = H-acceptor. [b]Symmetry code: (a) 1 – *x*, – *y*, – *z*; (c) 1/2 – *x*, 1/2 – *y*, – *z*; (d) 3/2 – *x*, 1/2 – *y*, – *z*; (e) *x*, – *y*, –1/2 + *z*. [c]Very weak interactions discussed in the text are included.

Table S3. Selected bond distances (Å) and angles (°) for **2**[a,b]

Cu(1)-O(1)	1.994(2)	Cu(1)-N(2)	1.988(3)
Cu(1)-O(2)	2.015(2)	Cu(1)-N(21)	1.975(2)
Cu(1)-O(6)	2.341(2)	Cu(1)-O(3Pa)	2.808(7)
Cu(2)-O(3)	1.948(2)	Cu(2)-N(23)	1.963(3)
Cu(2)-O(4)	1.994(2)	Cu(2)-O(1w)	2.432(3)
Cu(2)-N(22)	1.996(3)	Cu(2)-O(1P)	2.851(11)
Cu(3)-O(5)	2.256(2)	Cu(3)-N(26)	1.984(3)
Cu(3)-N(24)	1.997(3)	Cu(3)-N(27)	2.004(3)
Cu(3)-N(25)	2.030(3)		
O(1)-Cu(1)-O(2)	83.25(8)	N(21)-Cu(1)-O(6)	99.29(10)
O(1)-Cu(1)-O(6)	87.40(9)	N(21)-Cu(1)-N(2)	94.42(10)
O(2)-Cu(1)-O(6)	93.50(9)	O(1)-Cu(1)-O(3Pa)	76.03(19)
N(2)-Cu(1)-O(1)	94.86(9)	O(2)-Cu(1)-O(3Pa)	78.6(2)
N(2)-Cu(1)-O(2)	178.08(9)	O(6)-Cu(1)-O(3Pa)	162.3(2)
N(2)-Cu(1)-O(6)	86.66(10)	N(2)-Cu(1)-O(3Pa)	100.7(2)
N(21)-Cu(1)-O(1)	168.86(10)	N(21)-Cu(1)-O(3Pa)	96.18(19)
N(21)-Cu(1)-O(2)	87.44(9)		
O(3)-Cu(2)-O(4)	82.54(9)	O(4)-Cu(2)-O(1P)	83.0(3)
O(3)-Cu(2)-N(22)	87.64(10)	N(22)-Cu(2)-N(23)	95.68(11)
O(3)-Cu(2)-N(23)	174.02(11)	N(22)-Cu(2)-O(1w)	95.50(12)
O(3)-Cu(2)-O(1w)	83.90(11)	N(22)-Cu(2)-O(1P)	81.2(3)
O(3)-Cu(2)-O(1P)	76.4(3)	N(23)-Cu(2)-O(1w)	100.68(12)
O(4)-Cu(2)-N(22)	163.01(10)	N(23)-Cu(2)-O(1P)	99.1(3)
O(4)-Cu(2)-N(23)	93.02(10)	O(1w)-Cu(2)-O(1P)	160.1(3)
O(4)-Cu(2)-O(1w)	97.19(11)		
N(24)-Cu(3)-O(5)	101.40(10)	N(25)-Cu(3)-N(26)	89.81(11)
N(24)-Cu(3)-N(25)	89.07(11)	N(25)-Cu(3)-N(27)	177.21(14)
N(24)-Cu(3)-N(26)	160.64(12)	N(26)-Cu(3)-O(5)	97.84(10)
N(24)-Cu(3)-N(27)	91.71(13)	N(26)-Cu(3)-N(27)	90.34(14)
N(25)-Cu(3)-O(5)	87.09(10)	N(27)-Cu(3)-O(5)	90.14(13)

[a]Symmetry code: (a) = $-x$, $2-y$, $-z$. [b]Only bond distances and angles involving the major components of the disordered perchlorate anions are listed here.

Table S4. Hydrogen bond distances (Å) and angles (°) in **2**[a,b,c]

D-H···A	d(D-H)	d(H···A)	d(D···A)	<(DHA)
N(11)-H(11)···O(3)	0.86	2.00	2.809(3)	156.0
N(12)-H(12)···O(2)	0.86	2.60	3.345(3)	146.3
N(13)-H(13)···O(4w)	0.86	1.96	2.785(5)	161.2
N(14)-H(14)···O(2w)	0.86	1.98	2.828(4)	170.7
N(15)-H(15)···O(2P)	0.86	2.30	3.065(5)	147.4
N(16)-H(16)···O(4)	0.86	2.02	2.863(3)	165.3
N(17)-H(17)···O(3w)	0.86	1.75	2.600(9)	168.4
O(1w)···O(5Pd)			3.008(8)	
O(2w)···O(6)			2.893(4)	
O(3w)···O(8P)			3.216(9)	
O(4w)···O(12Pb)			2.921(16)	
O(4w)···O(1P)			3.124(15)	
C(6)-H(6C)···O(1)	0.96	2.42	3.100(5)	127.3
C(12)-H(12C)···N(23)	0.96	2.57	3.305(5)	133.9
C(27e)-H(27e)···O(11P)	0.96	2.41	3.36(2)	171.4
C(22a)-H(22a)···O(9P)	0.96	2.70	3.467(8)	117.1

[a]D = H-donor and A = H-acceptor. [b]Symmetry code: (a) – *x*, 2 – *y*, – *z*; (b) 1 – *x*, 2 – *y*, – *z*; (d) 1 – *x*, 2 – *y*, 1 – *z*; (e) 1 – *x*, 1/2 + *y*, 1/2 – *z*. [c]Very weak interactions discussed in the text are included.
